# Adaptation, effectiveness, and implementation of a digital intervention for healthcare workers with psychological distress

**DOI:** 10.1192/j.eurpsy.2026.10361

**Published:** 2026-07-31

**Authors:** R. Mediavilla, B. García-Vázquez

**Affiliations:** 1 Mental Health, Centro de Investigación Biomédica en Red; 2 Psychiatry, Universidad Autónoma de Madrid; 3 Psychiatry, Clinical Psychology, and Mental Health, Hospital Universitario La Paz, Madrid, Spain

## Abstract

**Introduction:**

Over the past half-decade, many surveys conducted in high-demand healthcare settings across Europe and globally have highlighted the psychological toll on healthcare workers (HCWs). Meta-analytic estimates suggest that approximately one-third of HCWs report symptoms consistent with depression and/or anxiety disorders. This persistent challenge underscores the need for scalable, evidence-based interventions that can be implemented across individual, organisational, and policy levels. The European Commission’s Best Practices Portal highlights a stepped-care programme of digital interventions designed to support mental health. This programme includes two interventions developed by the World Health Organization: *Doing What Matters in Times of Stress (DWM)* and *Problem Management Plus (PM+).*

**Objectives:**

We will (1) present the results of the adaptation process of DWM and PM+, (2) analyse their effectiveness in a sample of distressed healthcare workers in Spain, (3) describe the implementation process, and (4) evaluate the DWM’s implementation under real-world conditions.

**Methods:**

We will synthesize evidence from three complementary studies: a qualitative study identifying key problems and needs among HCWs in routine clinical practice, a multi-centre randomised controlled trial testing the effectiveness and implementation of DWM and PM+, and a pilot study that examined implementation outcomes of two versions of DWM under real world conditions.

**Results:**

The qualitative study showed that doctors’ and nurses’ narratives could inform the adaptation of the two eHealth interventions, with narratives from nearly 100 stakeholders shaping the final version of the programme. The randomized control trial showed that DWM and PM+ were more effective than enhanced care as usual in reducing anxiety and depressive symptoms when delivered in a stepped-care format. A mixed-methods process evaluation also showed favourable implementation outcomes. The pilot implementation study revealed high participation rates and high acceptance rates, appropriateness, and feasibility, among healthcare workers in a primary care centre in Madrid, Spain.

**Conclusions:**

These findings show that this stepped-care programme of scalable psychological interventions can be effectively implemented within routine healthcare services, with potential relevance for Spain, and other European countries with similar healthcare systems. The results highlight the continued relevance of evidence.based, digitally delivered stepped/care models and support their broader adoption by policy makers and health systems, reinforcing the value of the European Commission’s Best Practices protocol as a guide for sustainable strategies to support HCW’s mental health.

**Disclosure of Interest:**

None Declared

